# Unexpected Entrapment of Caravel Microcatheter in Percutaneous Chronic Total Coronary Intervention

**DOI:** 10.18295/squmj.3.2024.016

**Published:** 2024-05-27

**Authors:** Muhammad A. Sadiq, Adil B. Al Riyami, Muhammad A. Khatri, Hafidh Ba Omar

**Affiliations:** Department of Medicine, Sultan Qaboos University Hospital, Sultan Qaboos University, Muscat, Oman

A 60-year-old male patient with known hypertension, diabetes, and atrial fibrillation was electively admitted at a tertiary care hospital in Muscat, Oman, for his percutaneous coronary intervention (PCI) to the left anterior descending/diagonal 2 (LAD/D2) bifurcation chronic total occlusion (CTO).

A Caravel microcatheter (Asahi Intec Co., Tokyo, Japan) was passed down to the first septal perforator (S1) of LAD over a Runthrough guidewire (Terumo Corporation, Tokyo, Japan) and the ipsilateral collateral channel to LAD was marked by the contrast microinjection technique [Figure 1]. A Sion Black (Asahi Intec Co., Japan) guidewire was traversed through the ipsilateral collateral into the 2nd diagonal beyond the CTO. The Caravel microcatheter failed to negotiate the acute bend in the distal part of the first septal perforator and then the decision was made to exchange the Caravel microcatheter with a coil-based braided microcatheter in order to apply rotational push to overcome the bend. However, during removal, the tip of the Caravel microcatheter unexpectedly became trapped in the calcified ostial part of the first septal perforator (S1) of LAD [Figure 2]. After several failed gentle pulling efforts, the proximal shaft of the Caravel microcatheter was cut and passed a 6F guide catheter extension, GuideLiner (Teleflex, Morrisville, USA) to reach close to the origin of S1. The distal part of the microcatheter’s tip was detached and left behind in the proximal part of S1 [Figure 3]. There was a loss of flow in the first septal perforator. The patient, however, remained clinically and haemodynamically stable with no chest pain or electrocardiogram changes. The procedure was completed in antegrade by wire escalation technique leaving the detached tip of the microcatheter *in situ* [Supplementary Videoclip 1].

Microscopic examination of the retrieved microcatheter at Asahi Intec Co.’s lab (Tokyo, Japan) revealed approximately 2 mm of the distal part of the Caravel’s tip was dislodged. There was a scratch originating from the torn end of the tip suggesting that the tip of the Caravel microcatheter was hooked by a protruding calcium nodule [Figure 4].

Patient consent was obtained for the publication of this work.

## Comment

CTO interventions are challenging, marked by steep learning curves and lower success rates, due to the complexity of chronically occluded vessels.^1^ Microcatheters are indispensable in CTO interventions, providing essential flexibility and agility to navigate complex and tortuous coronary anatomy. They act as conduits for guidewires, significantly enhancing the procedure by offering better support to the CTO guidewire, improving penetration capacity, facilitating the reshaping of the guidewire tip, enabling easy guidewire exchanges and safeguarding the proximal vessel from injuries induced by stiff CTO guidewires.^2^^,^^3^ The Caravel microcatheter, with its thin-walled, hydrophilic design and tapered tip, is valued for its ability to smoothly navigate, especially through retrograde micro-channels.

Microcatheter over-torquing is the most common cause of entrapment, particularly when undue rotation is applied to microcatheters, including the Caravel, that are not designed for rotational use.^4^ In the current case, no rotational force was applied to the Caravel during its advancement in the septal channel. It was decided to remove it after its failure to navigate the acute bend in the distal part of the first septal perforator. However, while it was being removed, it unexpectedly became trapped in the calcified ostial part of the S1 segment of the LAD, most likely by a protruding speck of calcium in the angulated part of S1. Various techniques for retrieving entrapped microcatheters have been described. The pulling technique involves traction on the entrapped microcatheter, but strong traction can result in vessel dissection or perforation and may also cause fracture of the microcatheter tip, especially with softer microcatheters like the Caravel.^4^ The telescoping technique involves using a guide catheter extension, which is advanced over the microcatheter’s shaft after cutting its proximal end.^5^ This technique helps safeguard against potential dissection or perforation of the vessel during the retrieval of entrapped microcatheters. In the current case, despite the use of a combination of the pulling and telescoping techniques, the tip of the Caravel was fractured. The eventual successful management of the situation, opening the CTO through an antegrade approach and leaving the detached tip of the microcatheter *in situ*, demonstrated a pragmatic approach to an unforeseen complication. This case serves as an educational example of the technical difficulties encountered during CTO PCI, highlighting the need for careful planning, the availability of alternative strategies and the readiness to manage complications to ensure patient safety and procedural success.

## Supplementary Information



## Figures and Tables

**Figure 1:**
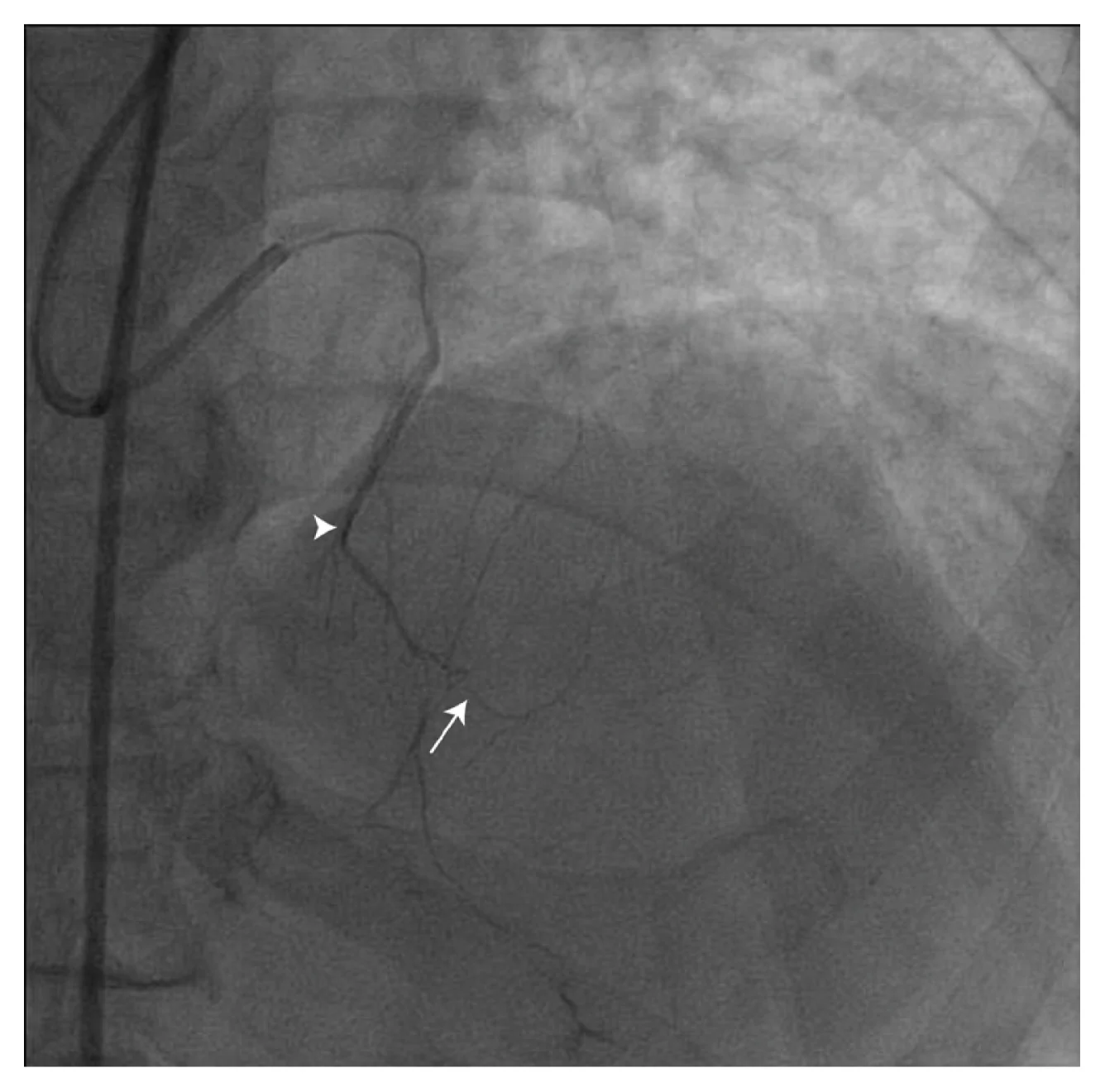
Contrast microinjection showing ipsilateral collateral to left anterior descending (arrow) and tip of Caravel microcatheter at acute bend in distal part of first septal perforator (arrowhead).

**Figure 2:**
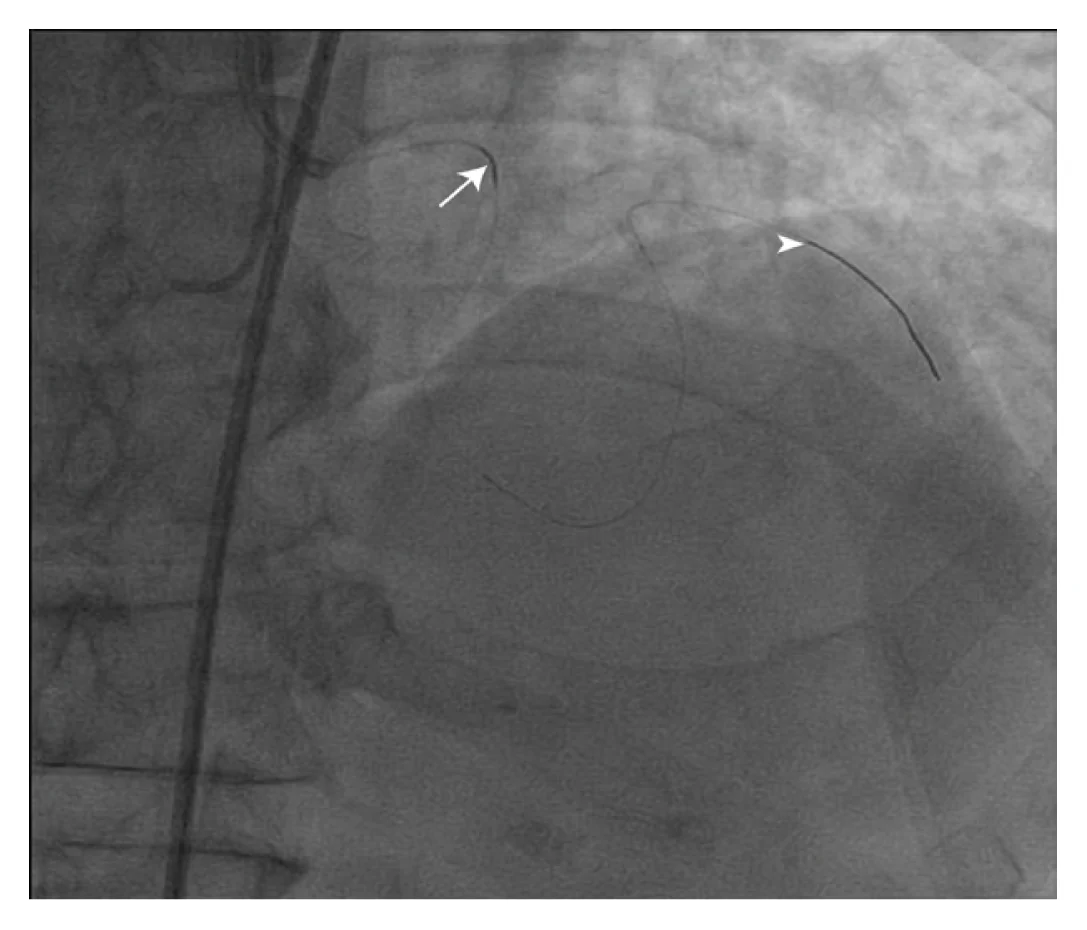
Image showing Caravel microcatheter’s tip entrapment position at ostium of first septal perforator (arrow) and Sion Black guidewire in 2nd Diagonal (arrowhead).

**Figure 3:**
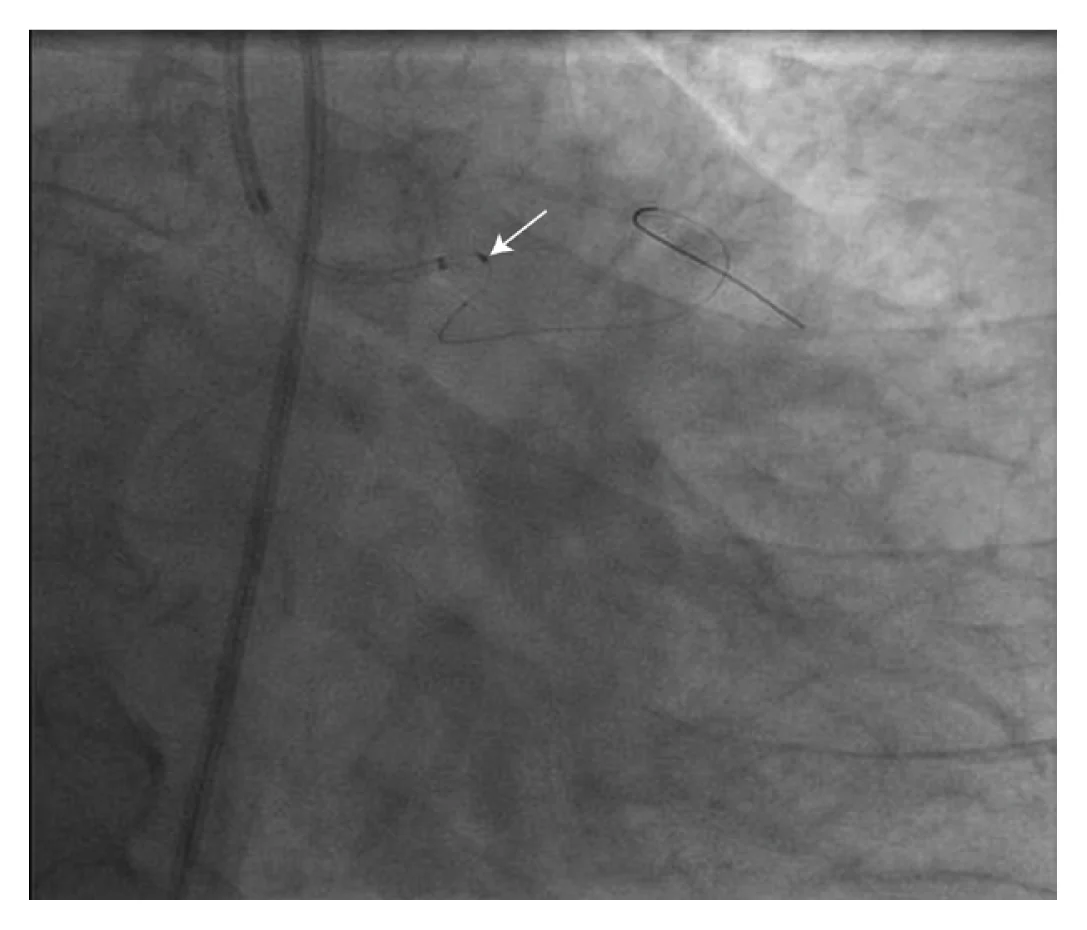
Anterior-posterior caudal view showing detached distal tip of Caravel microcatheter in first septal perforator (arrow).

**Figure 4:**
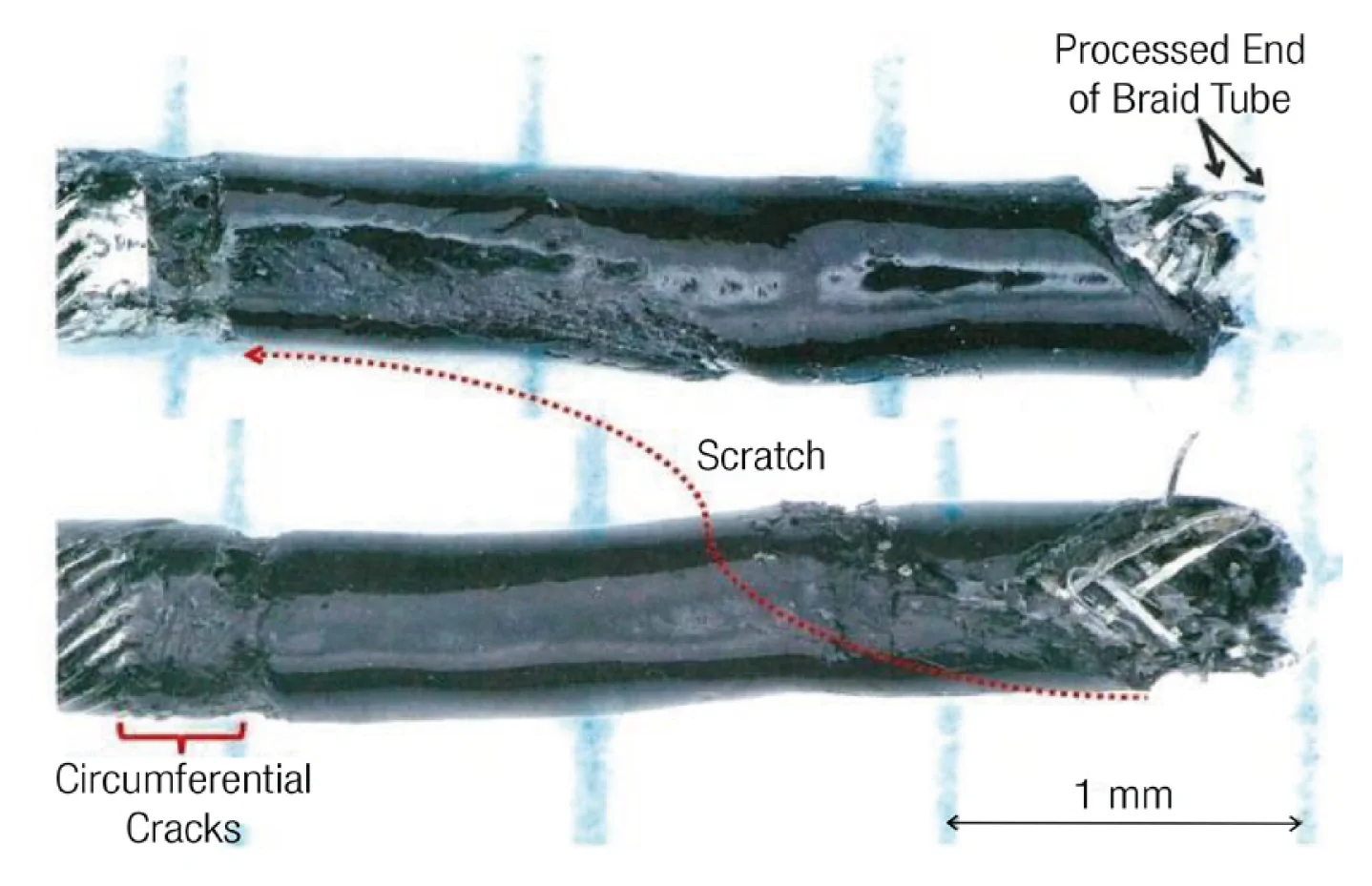
Microscopic examination of retrieved Caravel microcatheter showing circumferential cracks resulting from stretching of tip polymer at tip-shaft junction and scratch from the torn end of tip.
